# 1817. Characteristics and Outcomes in Patients with *Staphylococcus lugdunensis* Bacteremia Compared with *Staphylococcus aureus* and *Staphylococcus epidermidis* Bacteremia

**DOI:** 10.1093/ofid/ofac492.1447

**Published:** 2022-12-15

**Authors:** Satomi Yukawa, Taro Noguchi, Koh Shinohara, Yasuhiro Tsuchido, Masaki Yamamoto, Yasufumi Matsumura, Miki Nagao

**Affiliations:** Kyoto University Graduate School of Medicine, Kyoto, Kyoto, Japan; Kyoto University Graduate School of Medicine, Kyoto, Kyoto, Japan; Kyoto University Graduate School of Medicine, Kyoto, Kyoto, Japan; Kyoto University Graduate School of Medicine, Kyoto, Kyoto, Japan; Kyoto University Graduate School of Medicine, Kyoto, Kyoto, Japan; Kyoto University Graduate School of Medicine, Kyoto, Kyoto, Japan; Kyoto University Graduate School of Medicine, Kyoto, Kyoto, Japan

## Abstract

**Background:**

*Staphyococcus lugudnensis* (SL) is a coagulase negative *Staphylococcus* species with a potential to cause invasive infection. Few studies have evaluated characteristics and outcomes of SL bacteremia (SLB) compared with *Staphylococcus aureus* (SA) and *Staphylococcus epidermidis* (SE).

**Methods:**

We performed a single center retrospective case-control study of patients > =18 years of age with SLB with at least two sets of positive blood cultures at Kyoto University Hospital, Japan from January 2005 to December 2021. Patients who had SA bacteremia (SAB) with at least one set of positive blood culture and those who had SE bacteremia (SEB) with at least two sets of positive blood cultures were randomly selected in a 1:5:5 (SL:SA:SE) ratio during the same period. Characteristics and clinical outcomes of SLB were compared with those of SAB and SEB.

**Results:**

A total of 24 patients with SLB, 120 patients with SAB, and 120 patients with SEB were included. Patients’ median age (interquartile range) was 69 (60, 75), 68 (49, 77) and 65 (51, 73) in order of SL, SA, and SE. The rate of hospital-acquired infection in SL was similar to that of SA (54% vs 64%; p=0.86), lower than that of SE (54% vs 91%; p< 0.01). Rates of methicillin resistance and intravascular catheterization were higher in SE (83% and 92%, respectively). Patients with SLB were more likely to be on hemodialysis (25%). Rates of polymicrobial bacteremia and blood cancer was lower in SA (7% and 8%, respectively). Echocardiography was done for 14 patients in SL, lower than in SA (58% vs 82%; p=0.01). The rate of infective endocarditis (IE) (primary and secondary) was not significantly different among three groups (SL 8.5%, SA 6.5%, SE 2%). There was no significant difference in rate of metastatic lesions between SL and SA (17% vs 20%, p=0.9), statically lower in SE than in SL (17% vs 4%; p< 0.01). Seven-day mortality was similar between SL and SA (8% vs 7%; p=0.77), lower in SE than in SL (8% vs 0.5%; p=0.02). Thirty-day mortality in SL was not significantly different in other groups (SL 17%, SA 18%, SE 10%).

**Figure:**
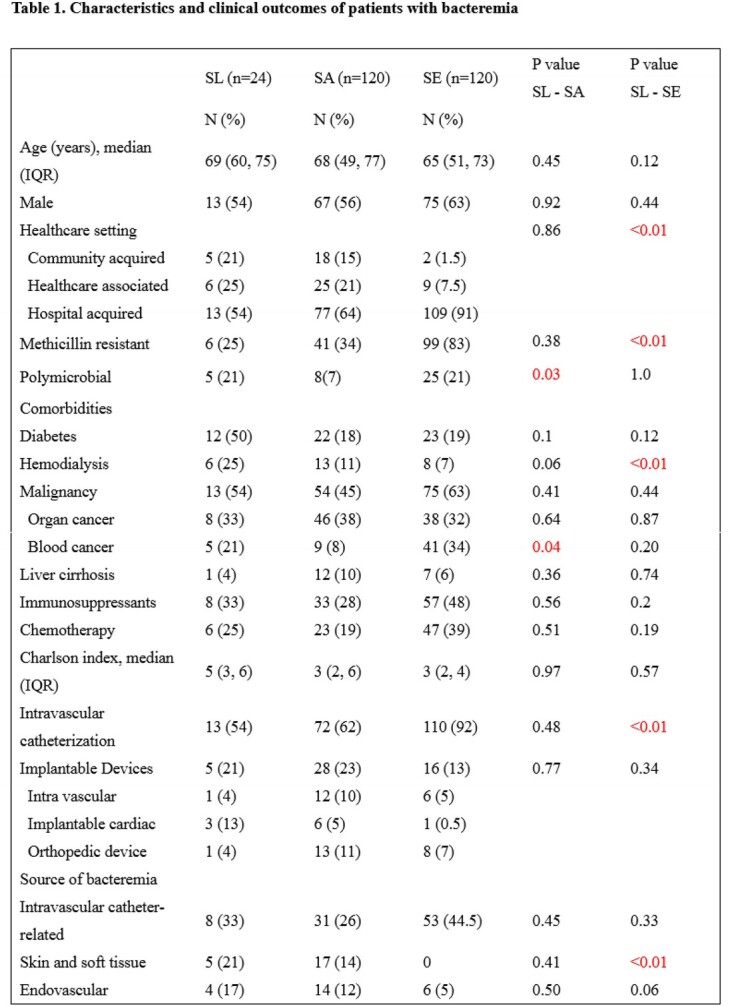

**Figure:**
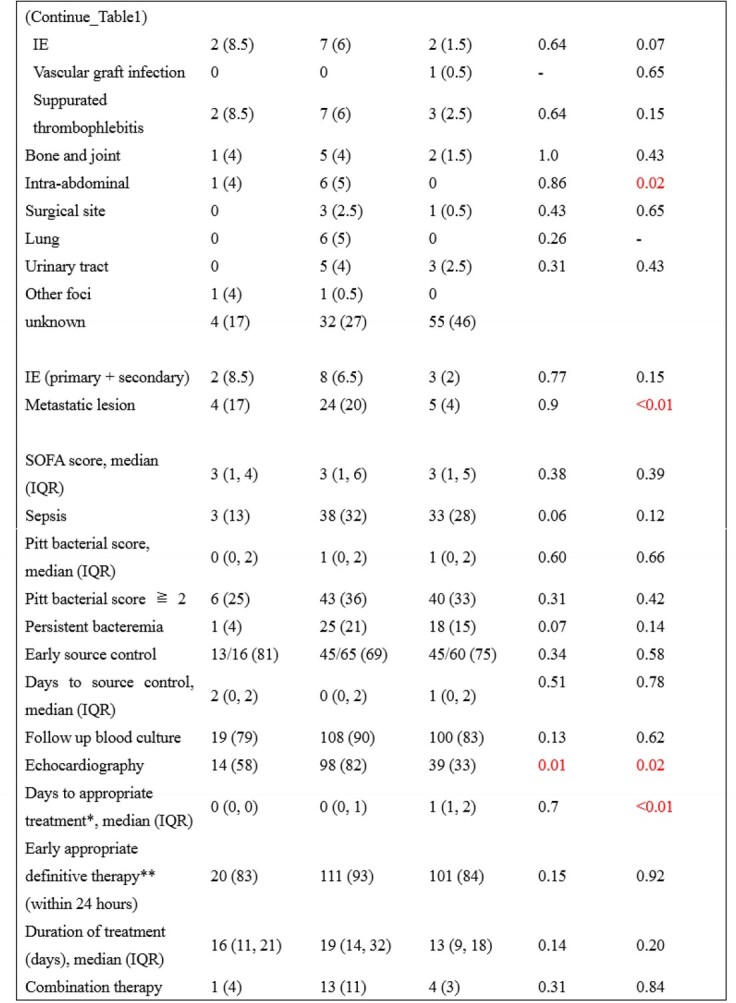

**Figure:**
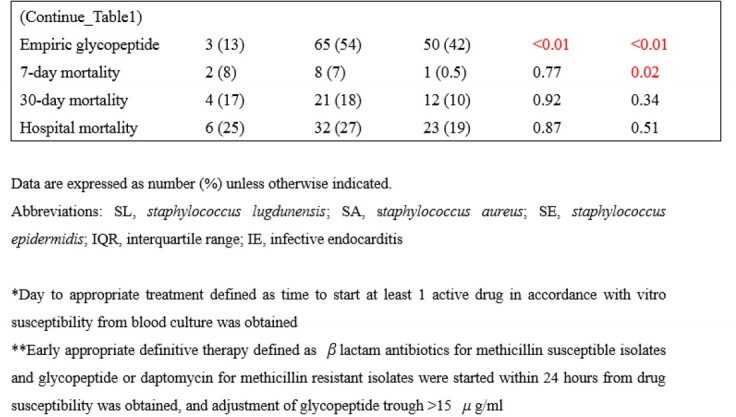

**Conclusion:**

The rates of metastatic lesions and 7-day mortality in SL were similar to that of SA, higher than that of SE. Appropriate evaluation and treatment that are recommended for SAB may be warranted for patients with SLB.

**Disclosures:**

**All Authors**: No reported disclosures.

